# Collection of Epithelial Cells from Rodent Mammary Gland Via Laser Capture Microdissection Yielding High-Quality RNA Suitable for Microarray Analysis

**DOI:** 10.1007/s12575-010-9026-8

**Published:** 2010-03-03

**Authors:** John N McGinley, Zongjian Zhu, Weiqin Jiang, Henry J Thompson

**Affiliations:** 1 Cancer Prevention Laboratory, Colorado State University, 1173 Campus Delivery, Fort Collins, CO 80523, USA

**Keywords:** Laser capture microdissection, Mammary epithelial cells, RNA quality

## Abstract

Laser capture microdissection (LCM) enables collection of cell populations highly enriched for specific cell types that have the potential of yielding critical information about physiological and pathophysiological processes. One use of cells collected by LCM is for gene expression profiling. Samples intended for transcript analyses should be of the highest quality possible. RNA degradation is an ever-present concern in molecular biological assays, and LCM is no exception. This paper identifies issues related to preparation, collection, and processing in a lipid-rich tissue, rodent mammary gland, in which the epithelial to stromal cell ratio is low and the stromal component is primarily adipocytes, a situation that presents numerous technical challenges for high-quality RNA isolation. Our goal was to improve the procedure so that a greater probe set present call rate would be obtained when isolated RNA was evaluated using Affymetrix microarrays. The results showed that the quality of RNA isolated from epithelial cells of both mammary gland and mammary adenocarcinomas was high with a probe set present call rate of 65% and a high signal-to-noise ratio.

## 1 Introduction

Rodent models of breast cancer continue to play a crucial role in discovering new approaches to treatment and prevention of the disease. Microarrays offer investigators the ability to screen thousands of genes per sample [1-5]. However, heterogeneous tissue may confound molecular analysis because it is currently impossible to discern which cells contribute which cellular constituents to a given tissue lysate. Laser capture microdissection (LCM) allows specific cell populations to be harvested, thus reducing the amount of biological noise while increasing the sensitivity of microarrays enabling detection of subtle differences in gene expression profiles among control and treatment groups [6]. Specific cell populations can be harvested from frozen or paraffin tissue sections using LCM [7-10]. However, RNA obtained from formalin-fixed paraffin-embedded tissue can be significantly degraded due to fragmentation and modification of the template through the addition of mono-methylol groups to the bases, thus interfering with RNA extraction and subsequent amplification [11-14]. Alternative methods of fixation and processing have been proposed for preservation of RNA in paraffin-embedded samples [15-17]. Nevertheless, they have not gained wide acceptance, and frozen tissue remains the gold standard for obtaining high-quality RNA.

**Figure 1:**
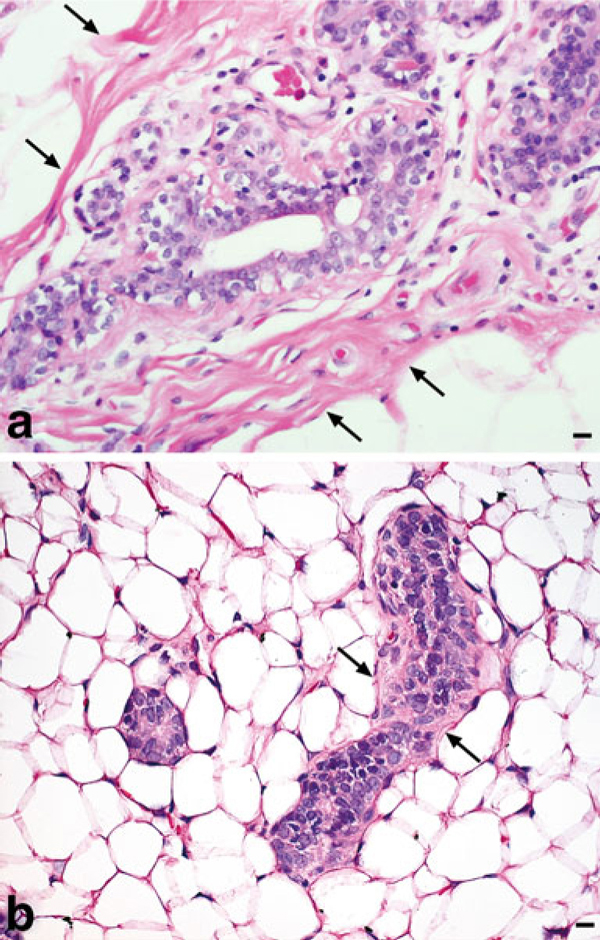
**Human breast vs. rat MG**. **a** Normal human breast ductal epithelium surrounded by thick fibrous connective tissue (*arrows*), H&E, ×400, *bar* = 10 μm. **b** Normal rat MG depicting ductal epithelium with a thin layer of connective tissue (*arrows*) surrounded by numerous adipocytes, H&E, ×400, *bar* = 10 μm.

Obtaining suitable frozen sections for LCM from lipid-rich tissues presents many challenges, and rat mammary gland (MG) presents specific obstacles due to the high adipose content and relative low abundance of fibrous connective tissue as compared to human breast (Figure 1). Capturing the limited number of epithelial cells available in each MG sample is a race against time in order to preserve RNA integrity. While there are a growing number of articles on the subject of LCM, few have reported optimized conditions and detailed hands-on procedures to obtain high-quality RNA suitable for microarray analysis from rodent MG that yields a high percent call rate when the RNA is arrayed on commercially available microarrays such as the Affymetrix platform [13,18-23]. Our laboratory spent over a year of developing, refining, and optimizing techniques necessary to overcome the inherent obstacles in rat MG prior to publication [24]. Colleagues have inquired regarding specific details of our approach to MG whole mount preparation, cryosectioning, and processing for LCM and RNA isolation. Therefore, it is our intent to describe in detail the techniques that resulted from addressing the following questions: (1) what is the best approach to obtain frozen sections of mammary adenocarcinoma (MA) and normal rat MG suitable for LCM; (2) can sections for LCM be stained without affecting RNA quality; (3) what is the ideal dehydration procedure for the preservation of RNA integrity; (4) how many LCM caps and laser fires per cap are necessary to produce a sufficient amount RNA for downstream gene array analyses; and (5) what is the maximum amount of time permitted for LCM per cap in order to maintain high-quality RNA yield?

## 2 Materials and Methods

Female Sprague–Dawley rats were obtained from Taconic farms (Germantown, NY) at 20 days of age (DOA). At 21 DOA, rats were given an i.p. injection of 50 mg 1-methyl-1-nitrosourea per kilogram body weight, Ash Stevens, Detroit, MI [25]. Animals were euthanized based on palpated tumor diameter ≤0.5 cm. A tumor was excised when its diameter reached ~0.5 cm or its volume reached ~62.5 mm^3^ and was immediately snap-frozen in liquid nitrogen. The contralateral abdominal inguinal MG chain was excised from the same animal, spread as a whole mount on transparency film (3M, St. Paul, MN., cat. no. PP2500), and lymph node (LN) chain marked on the underside of the film before snap freezing in liquid nitrogen. All samples were stored at -80°C. The work followed ethical guidelines approved by the Colorado State University Animal Care and Use Committee. One small frozen MA of relatively equal size was chosen from each animal for LCM in recognition that smaller tumors would have minimal necrosis, which adversely effects RNA quality. A representative area of frozen MG near the LN region in gland 4 was selected for LCM to serve as the source of reference (control) mammary epithelial cells.

**Figure 2:**
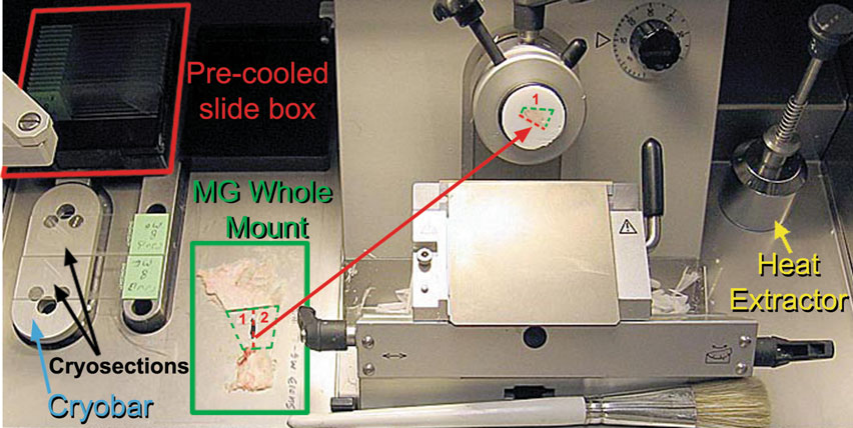
**Laser capture microdissection**. Cryosectioning chamber depicting MG whole mount bisected through the LN chain (*red dashed line*). Bisected MG halves were mounted on separate object holders and rotated at a slight angle with respect to the blade. The LN chain served as an anchoring point, making it easier to section MG with high adipose content.

Nuclease-free (NF) technique was used during all subsequent stages of sample handling. A heat extractor was used to both flatten and expedite freezing of the optimal cutting temperature (OCT) embedding media (Sakura Finetek, Torrance, CA., cat. no. 4583). MA and MG cryosections were cut at 7 and 10 μm, respectively (Leica Microsystems, Bannockburn, IL., cat. no. CM1850) and placed on plain glass slides (Surgipath Medical Ind., Inc., Richmond, IL., cat. no. 00330). Slides were placed on a Peltier cooled cryobar (-60°C) immediately after sectioning. Once frozen, slides were moved to pre-cooled plastic slide box (BD Biosciences, San Jose, CA., cat. no. 423843) located inside the cryo-chamber while additional sections were cut (Figure 2). A total of 25 slides were cut for each sample and total sectioning time was ≤20 min. Slides were stored at -80° until LCM could be performed.

**Figure 3:**
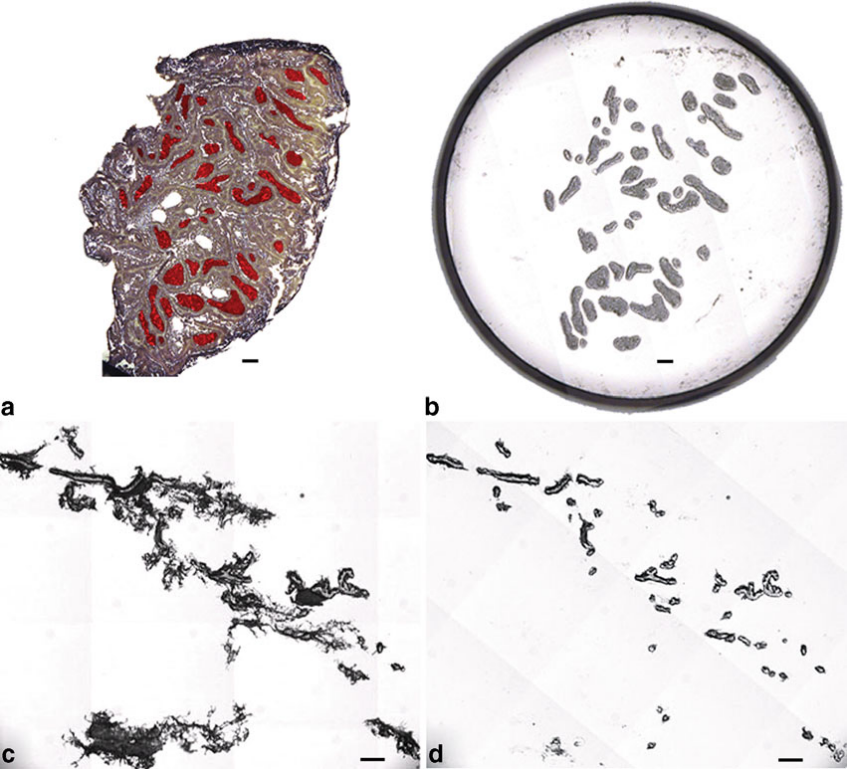
**a Unstained MA section showing AOIs marked in *red***. **b** Image of LCM cap showing captured epithelial AOIs. **c** LCM of normal MG depicting stromal debris attached to the macro cap. **d** Cap area from panel C after removal of stromal debris via adhesive note.

Plastic slide staining jars (Evergreen Scientific, Los Angeles, CA., cat. no. 240-5440-G8K) were cleaned with RNaseZap^®^ (Applied Biosystems/Ambion, Austin, TX cat. no. 9780), rinsed in DEPC-treated water, and allowed to air dry completely under a fume hood. Staining jars were placed in a plastic scintillation vial rack (VWR, West Chester, PA., cat. no. 66022-503) to prevent spillage. Slides were removed from the -80°C freezer and the slide box transported under dry ice to a chemical fume hood near the AutoPix^®^ LCM instrument (Molecular Devices, Sunnyvale, CA). Slides were removed from under dry ice one at a time, not allowed to thaw, and immediately dehydrated using NF HistoGene^®^ refill kit (Molecular Devices, cat. no. KIT0419) reagents according to the following modified protocol: 75% EtOH, H_2_0, 75% EtOH, 95% EtOH, and 100% EtOH all for 30 s each followed by xylene for 1 min and air drying for 1 min [24]. To maximally remove OCT, one brief H_2_0 rinse was adopted to shorten incubation time in 75% EtOH because the timing is critical for obtaining high quality of RNA. Slides were agitated vertically several times in each reagent to ensure reagent transfer and adequate dehydration. Each slide was immersed diagonally in the slide jar as opposed to using the plastic grooves provided, which allowed greater freedom of movement during agitation in each reagent. No more than four slides were passed through each set of reagents in order to minimize carryover. Each slide was quickly inspected to make sure that no residual xylene was present on the tissue or slide, which could damage the polymer on the surface of the LCM cap. Grossly visible folds or wrinkles present at the periphery of the tissue were removed using a sterile NF scalpel blade. In addition, a PrepStrip™ (Molecular Devices, cat. no. LCM207) static strip was applied to each tissue section to flatten and remove any loosely bound material that might interfere with proper seating of the LCM cap. Slides were placed on the AutoPix^®^ instrument one at a time and a CapSure^®^ macro cap (Molecular Devices, cat. no. LCM0211) was placed directly on top of the dehydrated tissue section and off center such that slightly more than half of the cap surface covered the tissue and the remainder covered a blank portion of the glass slide. Laser parameters were adjusted to ensure adequate wetting and subsequent capture. A small region of interest (ROI) comprising ≤30 microscopic field tiles was selected at ×10 and acquired as a static image. An Intuos digital tablet (Wacom, Vancouver, WA) was used to draw multiple areas of interest (AOI) on the static image intended for LCM (Figure 3a). The IR laser was fired capturing only the cells lying within the marked AOIs. The cap was placed on a blank portion of the glass slide and the entire cap area quickly imaged at ×4 magnification to verify cell collection (Figure 3b). Stromal debris was more prominent in captures from MG than in MA (Figure 3c). Debris was removed by lightly stamping the cap on the tacky portion of a clean adhesive note (3M, cat. no. MMM654YW) three times, which removed debris but left captured epithelium intact (Figure 3d) [10]. The cap was placed on a 0.5-mL microfuge tube (Applied Biosystems, Foster City, CA., cat. no. N8010611) containing 30 μL of PicoPure^®^ RNA extraction buffer (Molecular Devices, cat. no. KIT0204), inverted, and gently tapped on the bench top several times to ensure adequate coverage across the surface of the polymer. A total of four caps from each MA and eight caps from each MG were collected.

### 2.1 RNA Extraction

**Figure 4:**
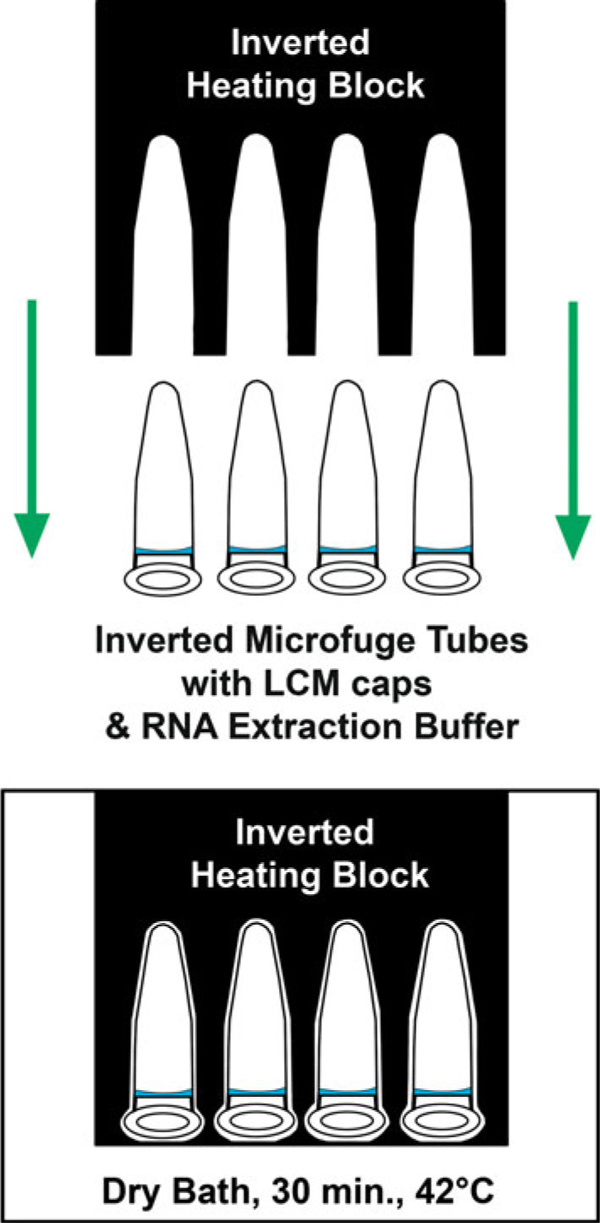
**Proper inverted orientation of microfuge tubes and heating block**. Microfuge tubes were kept inverted during initial RNA extraction, ensuring adequate coverage of extraction buffer across the surface of the LCM cap.

A dry bath and heating block were pre-warmed to 42°C. The heating block was removed and inverted tubes were quickly placed at the bottom of the dry bath. The heating block was inverted, placed on top of the tubes to ensure adequate heat retention, and incubated at 42°C for 30 min (Figure 4). Tubes were centrifuged for 2 min at 800×*g* to collect cell extract and stored at -80°C until the remaining portion of the RNA extraction procedure could be completed. The PicoPure^®^ kit (Molecular Devices, cat. no. KIT0204) was used to extract RNA. Briefly, RNA purification columns were prepared by adding 250 μL of conditioning buffer onto the purification column filter membrane and incubating for 5 min at room temperature. The column collection tube was centrifuged at 16,000×*g* for 1 min. The cell extract was treated with 30 μL of 70% EtOH and mixed well. The cell extract EtOH mixture was pipetted into the pre-conditioned purification column and centrifuged for 2 min at 100×*g* to bind RNA to the column, immediately followed by a centrifugation at 16,000×*g* for 30 s to remove flow-through. Cell extracts isolated from multiple LCM caps of each sample, four MA and eight MG caps, respectively, were processed through the same column to increase RNA yield. Wash buffer 1 (WB1, 100 μL) was added to the purification column and centrifuged for 1 min at 8,000×*g*. DNase working solution was diluted by adding 5 μL DNase I stock solution DNase, RNase-Free DNase Set (Qiagen, cat. no. 79254) to 35 μL buffer RDD (provided with NF Set) and mixed by gently inverting. Forty microliters of the DNase working solution was added directly into the purification column membrane and incubated at room temperature for 15 min. The DNase was washed by adding 40 μL PicoPure^®^ RNA Kit WB1 to the purification column membrane and centrifuged at 8,000×*g* for 15 s, followed by a second buffer wash, 100 μL wash buffer 2 (WB2), and centrifuged for 1 min at 8,000×*g*. The column was washed again by adding 100 μL WB2 into the purification column and centrifuged for 2 min at 16,000×*g*. The column was inspected for any residual WB and if found was re-centrifuged at 16,000×*g* for 1 min. The column was transferred to a new 0.5-mL microcentrifuge tube provided in the kit. RNA was eluted by adding 11 μL of elution buffer directly onto the membrane of the purification column and incubated for 1 min at room temperature followed by centrifugation of the column for 1 min at 1,000×*g* and 1 min at 16,000×*g*. The isolated RNA was stored at -80°C.

### 2.2 Total RNA Quantity and Integrity

The concentration of each total RNA sample was determined using a NanoDrop^®^ ND-3300 Fluorospectrometer (NanoDrop, Wilmington, DE). The assay measured fluorescence of RiboGreen dye at 525 nm following excitation at 470 nm. RNA concentration was calculated based on a standard curve. The integrity of total RNA samples was examined by Experion (Bio-Rad, Hercules, CA, cat. no. 700-7001) using the Experion RNA HighSens analysis kit (cat. no. 700-7105).

### 2.3 RNA Amplification and Labeling for Microarray

The Ovation™ Biotin RNA amplification and labeling system was used for amplification and labeling (NuGEN Technologies, Inc., San Carlos, CA., cat. no. 2300). The Ovation™ Biotin System is powered by Ribo-SPIA Technology, a rapid, simple, and sensitive RNA amplification process. A detailed protocol is described in the user's guide kit and was used without modification as reported previously [24].

### 2.4 Hybridization, Washing, and Staining of the Affymetrix GeneChip^®^ Rat Genome 230 2.0 Arrays

Hybridization was performed by incubating 200 μL of the above hybridization cocktail to the Affymetrix GeneChip^®^ arrays (Affymetrix, Inc., Santa Clara, CA). Hybridization occurred at 45°C for 16 h using a GeneChip^®^ Hybridization Oven 640 (Affymetrix). After hybridization, the hybridization solutions were removed and the arrays washed and stained with streptavidin-phycoerythrin using a GeneChip^®^ Fluidics Station 450 (Affymetrix). Arrays were read at a resolution of 2.5 to 3 Am using the GeneChip^®^ Scanner 3000 (Affymetrix).

### 2.5 Statistical Analyses

Raw expression values from 42 chips of the Affymetrix Rat Genome 230 2.0 were generated using GeneChip^®^ Operating Software (Affymetrix). All analyses were performed using Systat statistical analysis software, version 12 (Systat Software, Inc., San Jose, CA)

## 3 Protocol

### 3.1 Necropsy

1. Harvest all tissues as quickly as possible, i.e., <4 min from time of killing.

2. Excise the abdominal–inguinal MG chain from one side of the animal and spread onto a pre-cut piece of transparency film, mark the lymph node chain on the bottom of the film using a permanent marker, place in a 4 × 6-in. heat seal bag (do not seal), and snap freeze in liquid nitrogen without embedding in OCT.

3. Excise tumors, place in cryovials, and snap freeze in liquid nitrogen.

4. Store tissues at -80°C until ready to cut frozen sections.

### 3.2 Cryosectioning

1. Clean the cryostat chamber, including the knife blade holder with 200 proof ethanol.

2. Remove a disposable microtome blade previously stored in 200 proof ethanol, allow to air dry, and insert into the cryostat knife blade holder.

3. Set cryostat to optimized temperature for tissue type and allow time to equilibrate, e.g., -24°C for mammary tumor or -30°C for MG.

4. Remove one tissue at a time from -80°C freezer, transport under dry ice, and place the sample in the cryostat.

5. MG: Remove film-mounted gland from bag, bisect the gland longitudinally through the marked lymph node chain using a clean scalpel or razor blade, then cut above the superior node and below the inferior node to obtain two halves of mammary tissue.

6. Using a room temperature object holder, dispense enough OCT on the holder to adequately cover the surface and place it on the cryobar to cool.

7. Once the OCT begins to turn opaque, place the sample on top of the OCT, dispense additional OCT to cover the sample, and place a heat extractor on top to flatten and freeze the OCT quickly.

8. Place the object holder in the chuck of the cryotome and rotate. MG tissue should be rotated with the edge of the lymph node chain angled to the knife blade.

9. Adjust cutting depth to 7 μm for tumor or 10 μm for MG.

10. Cut sections and place on clean, room temp, plain glass slides (orient sections in the middle toward the lower half of the slide).

11. Immediately place the slide on the Peltier cooled portion of the cryobar to freeze as quickly as possible.

12. Once frozen, place the slide in a pre-cooled, clean, small plastic slide box inside the cryo chamber. One box per sample.

13. Cut additional sections for use as replicates to minimize freeze/thaw problems, but limit total sectioning time to <20 min.

14. Return the sample and slide box of cut sections to -80°C until ready for dehydration and LCM.

### 3.3 Dehydration

1. Fill pre-cleaned (nuclease-free) plastic staining jars with Histogene^®^ reagents and order as follows: 75% ethanol (EtOH), H_2_0, 75% EtOH, 95% EtOH, 100% EtOH, xylene.

2. Remove one sample at a time from -80°C and transport under dry ice to the chemical fume hood.

3. Remove one slide from the slide box; do not thaw and immerse immediately in 75% EtOH for 30 s. Transfer the slide from one reagent to the next in the order listed above and incubate for 30 s in each reagent with the exception of xylene (1 min). A pair of forceps should be used to agitate the slide vertically in each reagent solution for the duration of each incubation period in order to ensure adequate reagent transfer. Regent should be drained from the slide prior to immersion in subsequent reagents to minimize cross-contamination. No more than four slides should be used in one set of reagents. The slide should be air-dried under the fume hood. Rapid reciprocal movement of the slide under the hood will help facilitate the air drying process.

4. Inspect the slide for any folds, wrinkles, or debris at the edges of the tissue section and remove with a clean scalpel blade.

5. Place a PrepStrip™ on top of the dried tissue section, rub index finger or thumb over the length of the strip three to four times, and peel the strip off of the section to remove any debris that might interfere with seating of the laser capture microdissection (LCM) cap.

6. Proceed immediately with LCM.

### 3.4 Laser capture microdissection

1. Load the dry slide in the LCM instrument, focus, and acquire a road map image of the tissue section.

2. Move the red box on the road map image to the desired location, right click, and choose "place cap at region center." The cap should be position such that only one half to three fourths of the cap surface covers the tissue section with the remainder covering a blank portion of the glass slide.

3. Using the ×10 objective, bring the tissue section into focus then move the active window to a blank portion of the cap.

4. Locate and focus the laser then test fire to verify adequate wetting (black ring with a clear center).

5. Default laser settings for pulse (1,500 μs), hits (1) and delay (0) should be adequate for 7-μm tumor sections, but the power level may need to be increased from 60 to 90 mW. Thicker MG sections (10 μm) may require increasing the number of hits from 2 to 20, and the delay should be increased from 0 to 10 μs.

6. Test fire the laser and set the spot size.

7. Draw a ROI on road map image and acquire the region as static image (no more than 30 image tiles).

8. Use a digital tablet in conjunction with the free-hand line and polygon tools to quickly draw or mark multiple areas on the static image intended for capture. When finished, right click and choose capture. Repeat the ROI selection and marking process on different areas within the cap as time permits.

9. Unload the cap and stamp three times on tacky portion of a clean self-adhesive note to remove debris.

10. Place the cap on a blank portion of the glass slide and reacquire the road map image.

11. Using the ×4 objective, draw a ROI around the cap and acquire as a static image. Inspect the image to verify successful capture of epithelial cells.

12. Open and pull the lid completely off of the microfuge tube filled with 30 μL of RNA extraction buffer (PicoPure^®^ RNA isolation kit).

13. Remove the cap from the slide and seat the cap on the microfuge tube, being careful not to crack the tube.

14. Invert the tube and tap on the bench top several times to ensure the buffer covers the surface of the cap.

15. Total time from start of dehydration procedure to extraction buffer should be ≤30 min.

### 3.5 RNA Extraction

1. Place inverted tubes in a pre-warmed heating block and incubate at 42°C for 30 min.

2. Remove the tubes from the heating block and centrifuge at 800×*g* for 2 min.

3. Store tubes at -80°C or proceed with remainder of extraction procedure.

4. Pre-condition the RNA purification column using 250 μL of conditioning buffer and incubate for 5 min at room temp.

5. Centrifuge the column at 16,000×*g* for 1 min.

6. Pipette 30 μL of 70% EtOH into the cell extract from RNA extraction and mix by pipetting up and down; do not centrifuge.

7. Pipette the cell extract and EtOH mixture into the pre-conditioned purification column.

8. Centrifuge for 2 min at 100×*g* followed by centrifugation at 16,000×*g* for 30 s to bind RNA to the column.

9. Pipette 100 μL WB1 into the purification column and centrifuge for 1 min at 8,000×*g*.

10. DNase treatment (use RNase-Free DNase Set (Qiagen, catalog no. 79254).

11. Pipette 5 μL DNase I stock solution into 35 μL Buffer RDD (provided with RNase-Free DNase Set) and mix by gently inverting.

12. Pipette the 40 μL DNase incubation mix directly into the purification column membrane and incubate at room temperature for 15 min.

13. Pipette 40 μL PicoPure^®^ RNA Kit WB1into the purification column membrane and centrifuge at 8,000×*g* for 15 s.

14. Pipette 100 μL (WB2) into the purification column and centrifuge for 1 min at 8,000×*g*.

15. Pipette another 100 μL WB2 into the purification column and centrifuge for 2 min at 16,000×*g*.

16. Check the purification column for any residual WB. If WB remains, re-centrifuge at 16,000×*g* for 1 min.

17. Transfer the column to a new 0.5 mL microcentrifuge tube provided in the kit.

18. Pipette 11 μL of elution buffer (EB) directly onto the membrane of the column and incubate for 1 min at room temperature.

19. Centrifuge the column for 1 min at 1,000×*g* to distribute EB in the column.

20. Centrifuge for 1 min at 16,000×*g* to elute RNA. The isolated RNA is now ready for use in downstream applications. The entire sample may be used immediately or stored at -80°C until ready to use.

## 4 Results and Discussion

### 4.1 Cryosectioning

The high adipose content and low abundance of epithelial cells and connective tissue present in normal MG made the process of obtaining thin, flat, intact cryosections suitable for LCM a difficult challenge. While MA sections cut very nicely at 7 μm, MG sections had to be cut at 10 μm due to the high adipose content. MG cryosections cut at <10 μm produced fragmented sections or no sections at all, while sections >10 μm were too thick for LCM. Other instruments, which offer a UV cutting laser including the Veritas and Arcturus^XT^ systems, are able to microdissect tissue >10 μm. However, The Pixcell and Autopix^®^ instruments do not have a UV laser to physically cut out AOIs and rely solely upon the adhesive properties of the polymer to overcome the shear force necessary to separate the AOIs from the surrounding tissue and glass slide. The thicker the tissue section, the greater the shear force required. It has been reported that large microdissected areas captured via IR or UV show similar 18/28-s peak profiles, while the profiles of small areas, e.g. single cells microdissected using a UV cutting laser, show considerable RNA degradation [26]. Normal ductal mammary epithelium is only one to two cell layers in thickness and would be subject to much higher levels of RNA damage than large areas of MA captured by UV. Conversely, the IR laser only reacts with polymer coating on the cap and does not harm the underlying tissue irrespective of the area captured.

MG sections required colder cutting temperature than MA sections, -30°C and -24°C, respectively. In many cases, the normal MG tissue disintegrated upon contact with the microtome knife blade. However, we found that bisecting the MG through the long axis of the LN chain and embedding the tissue with the LN chain angled to the edge of the knife blade created an anchoring point, which aided in cryosectioning (Figure 2). An early attempt was made to preserve RNA in tissue harvested at necropsy by immersing the tissue in RNAlater^®^ (Applied Biosytems/Ambion, cat. no. AM7024). However, the chemical components of this product greatly interfered with OCT embedding and subsequent cryosectioning. The best results were obtained when the tissue was snap-frozen in liquid nitrogen immediately after excision at necropsy, stored at -80°C, and, after being embedded in OCT, cryosectioned within 20 min then quickly stored at -80°C.

### 4.2 Staining, Dehydration, and LCM

**Figure 5:**
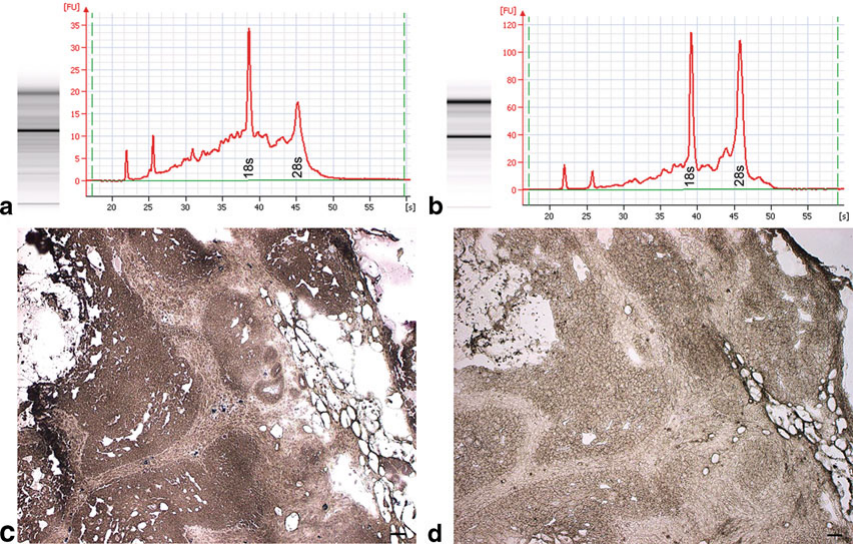
**Stained tissue vs. unstained tissue**. **a** Electropherogram of LCM acquired epithelia from Histogene^®^-stained MA showing significant rRNA degradation. **b** Electropherogram of LCM acquired epithelia from unstained MA showing high-quality rRNA. **c** Histogene^®^-stained MA. **d** Unstained MA.

The Histogene^®^ kit utilizes aqueous toluidine blue as the staining component, which requires an additional water rinse to remove excess stain prior to dehydration. Initial testing using stained sections revealed significant RNA degradation (Figure 5a). Since nucleases are active in aqueous solutions and it is critical that the time spent in such solutions be kept to a minimum in order to avoid RNA degradation, we modified the recommend protocol to determine if removal of the staining step would increase RNA quality. Unstained sections yielded higher quality RNA in the form of higher rRNA 28/18 s ratios (Figure 5b). The total time spent in aqueous solution using the standard Histogene^®^ method is 80–90 s compared to our modified unstained method of 30 s. A single water rinse was required in the unstained method to completely remove the OCT embedding medium after fixation in 75% EtOH. In addition, the amount of time in xylene and air drying were reduced from 5 to 1 min each. Given the shortened incubation times along with the small volume of reagents, slide forceps were used to provide continued vertical agitation of the slides in each vial in order to facilitate reagent transfer during the dehydration process. Slides were passed through reagents one at a time, and no more than four slides were ever passed through the same set of reagents in order to avoid excessive reagent carryover.

In theory, slides could be dehydrated and stored at -80°C with desiccant, possibly extending the storage life of cut sections. However, sections would still need to be dehydrated again prior to LCM in order to remove any condensation that may form on the slide as a result of contact with ambient air. Humidity conditions existing in laboratory environments can be variable depending upon geographic location. Successful LCM requires that tissue sections be absolutely dry. If any moisture is present in the tissue, the IR laser will react with the water molecules, causing the tissue to heat, thus degrading the RNA. Moreover, according to Espina et al. [10], complete dehydration is necessary to minimize the adhesive forces between the slide and tissue. Dehydrating sections once was more efficient, saving both time and reagents, and ensured that the tissue was as dry as possible prior to LCM.

An argument could be made for the removal of all H_2_0 steps in the dehydration procedure: that it serves no useful purpose except to remove OCT at the periphery of the tissue and exposure to aqueous environment increases the risk of RNA degradation, which should be avoided. While it is true that OCT remains at the periphery of the tissue and does not penetrate, it can interfere with proper seating of the cap if it is not removed from the section. The LCM cap is typically offset on the tissue section, i.e., a portion covers the tissue of interest while the remainder covers a blank portion of the glass slide. This is necessary to perform test fires of the laser in order to ensure adequate wetting, i.e., polymer to tissue/slide contact. This procedure must be performed on each new cap and every time the cap is moved to a different location. Laser settings will likely need to be adjusted in order achieve a similar laser spot size. Accurate measurement of the spot size is key in providing the computer the necessary *X*–*Y* coordinates in conjunction with degree of overlap to ensure capture of contiguous AOI. In addition, caps which cover most or all of the tissue run a greater risk of being placed on an area that may have variation in thickness, e.g., folds or wrinkles that will definitely interfere with proper seating. If the cap is not seated properly, this causes inconsistencies in the way the polymer contacts the tissue, which can lead to a phenomenon known as polymer depletion described by Espina et al. [10]. While immersion in graded ethanol, e.g., 75% EtOH, could remove OCT from the section, it would require extended incubation time in the reagent. Early attempts at bypassing the H_2_0 step altogether required aggressive agitation of the slide in the reagent and resulted in only partial removal. OCT could be removed by scraping the peripheral area(s) with a razor blade or scalpel. However, close visual inspection and physical removal near the section would be a time-consuming process, not to mention the possibility of contaminating the surface of the tissue section with dried flakes of OCT media. Dehydration and timing are critical. In the interests of efficiency and reproducibility, it was decided to keep one brief H_2_0 rinse rather than chance incomplete removal of the OCT.

An argument could also be made for substituting aqueous toluidine blue with an alcoholic stain. The intent was to optimize the original Histogene^®^ procedure [19] for use with mammary tissue sections by reducing incubation times or eliminating reagents. Therefore, only the staining reagent provided in the Histogene^®^ kit was used. Alcoholic-based versions of toluidine blue or nuclear fast red may fare better than aqueous stains. However, the authors sought to challenge the unwritten rule of pathology that tissue must be stained in order to visualize target cells of interest. Granted that while the use of unstained sections may not be applicable to all tissue types, ductal and lobular mammary epithelium are easily discernable in unstained sections, and stromal bands of connective tissue streaming through MA sections provide adequate contrast in order to differentiate epithelium from stromal component. Staining and subsequent differentiation lengthen the time necessary for adequate dehydration of the tissue, thereby increasing the risk of RNA degradation. The goal was to keep the modifications simple while maintaining the highest level of RNA integrity possible. Visualization of unstained sections (Figure 5d) was enhanced by lowering the light intensity on the Autopix^®^ instrument and was fairly comparable to that obtained in stained tissue (Figure 5c). Tiled images of H&E sections were used as guide maps for more challenging specimens.

### 4.3 LCM Time Course

**Figure 6:**
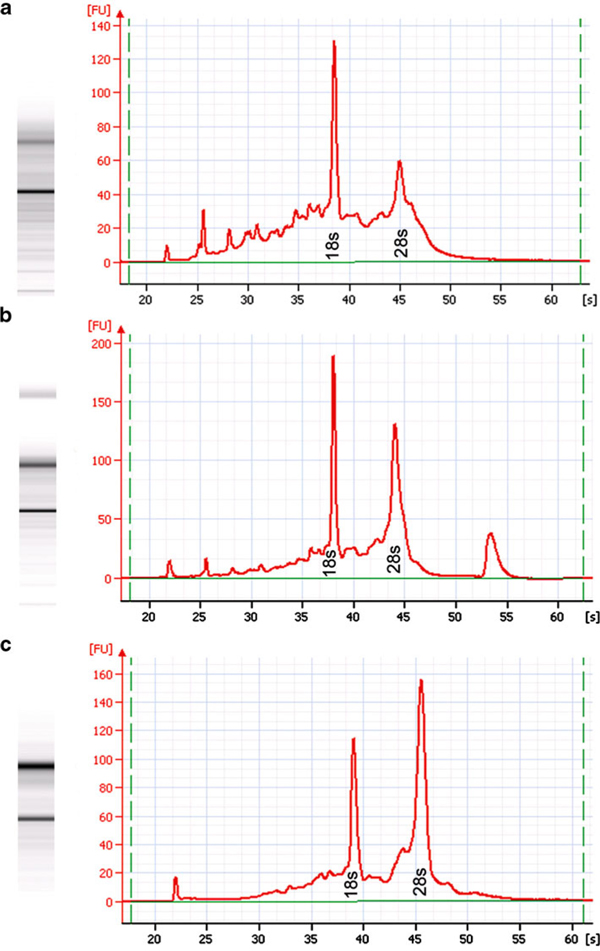
**Electropherogram comparison of LCM time course samples from unstained MA**. **a** LCM duration of 2 h. **b** LCM duration of 1 h. **c** LCM duration of 30 min. RNA quality determined by Bio-Rad Experion.

To evaluate the impact of LCM duration on RNA quality, samples were collected over 2 h, 1 h, and 30 min, respectively. The 2-h LCM sample showed significant RNA degradation (Figure 6a): RNA area = 1,550.1, concentration = 6,949 pg/μL, rRNA ratio (28/18 s):0.9, and RIN = 6.2. The 1-h LCM sample showed moderate RNA degradation (Figure 6b): RNA area = 1,430.8, concentration = 5,373 pg/μL, rRNA ratio (28/18s):1.3, and RIN = 7.7. The 30-min LCM sample exhibited minimal RNA degradation (Figure 6c): RNA area = 1,258.5, concentration = 51,443 pg/μL, rRNA ratio (28/18 s):1.7, and RIN = 8.0. These observations are consistent with the fact that RNA degradation is a time-dependent process. The highest quality results were obtained when the entire LCM process was ≤30 min, i.e., from the point the slide was removed from dry ice and immersed in 75% EtOH until captured cells were placed in extraction buffer; thus, LCM should be performed as quickly as possible to minimize RNA degradation.

### 4.4 RNA Quantity

RNA quality and quantity were determined using an Experion (Bio-Rad) automated electophoresis instrument and quantity data were validated by Nandrop^®^ ND-3300 fluorospectrophometer (RiboGreen). RNA quantity for both MA and MG were found to be highly correlated between the Experion and Nanodrop^®^ (Table 1). Four caps for tumor (mean cell count per cap = 6,202.2 ± 223.2) and eight caps for MG sections (mean cell count per cap = 994.2 ± 42.5) were necessary to provide sufficient amounts of RNA for downstream gene array analysis using the Ovation™ Biotin RNA amplification and labeling system.

**Table 1:** RNA yield and LCM cell estimate

Tissue	**Experion**^**a **^**(ng/μL)**	NanoDrop (ng/μL)	Total RNA (ng)	Laser fires per cap	**Cell Count per cap**^**b**^	**Cell Count per sample**^**c**^
MA (*n* = 27)	7.9 ± 0.8 (2.1, 16.7)	8.6 ± 0.8 (2.3, 17.8)	86	3,277.9 ± 141.0 (778.0; 8,212.0)	6,202.2 ± 223.2 (1,182.6; 12,511.5)	24,808.7 ± 1,270.2
MG (*n* = 27)	4.5 ± 0.4 (1.8, 10.4)	4.9 ± 0.5 (1.8, 14.5)	49	785.6 33.0 (61.0; 2,190.0)	994.2 ± 42.5 (69.5; 2,678.2)	8,192.2 ± 728.3

^a^Values are means ± SEM (min, max). Good correlation was observed between Experion and NanoDrop results obtained from both MA (*r* = 0.88, *p* < 0.01) and MG (*r* = 0.83, *p* < 0.01). Cell estimates were calculated based on the formula developed by Espina et al. [10]. Spot sizes ranged from 20 to 40 μm for MA and 10 to 15 μm for MG. A typical cell diameter of 7 μm was used for all rat mammary epithelium, percent overlap was kept constant at 40%, and 95% efficiency was assumed

^b^Because of limitations in the amount of time that could be spent collecting cells on each cap without RNA degradation and the dispensed nature of cell distribution within MG, there were fewer cells per MG cap and more variability in cell count per cap

^c^A total of four caps were dissected each MA and eight caps from each MG specimen

### 4.5 RNA Quality as Assessed by Microarray Quality Control Statistics

#### Average Background and Noise Values

Affymetrix QC statistics specify that average background values should range from 20 to 100 [27-29]. A total of 42 RNA samples, 21 from MG and 21 from MA, were evaluated. The average background value was 51.9 with a range of 50.1–53.4 (Table 2). As written in the Affymetrix technical manual, "the noise measurement is based on the pixel-to-pixel variation of probe cells on a GeneChip^®^ array, which comes from electrical noise of the scanner and sample quality" [30]. The same scanner was used for the analysis of all chip arrays. The acceptable noise generally should be <5, and our results were <3 (Table 2).

**Table 2:** Quality control of 42 GeneChips^®^^a^

Item	MG	MA	Overall	Expected
Background	55 ± 4	49 ± 3	52 ± 2	<100
Noise	2.3 ± 0.2	2.1 ± 0.2	2.2 ± 0.1	<5
Percent present (%)	65 ± 0.6	65 ± 0.8	65 ± 0.5	>35
3'/5' *dap*	2.0 ± 0.7	1.7 ± 0.5	1.8 ± 0.4	<3
3'/5' *lys*	1.0 ± 0.2	2.3 ± 0.6	1.7 ± 0.3	<3
3'/5' *phe*	3.3 ± 0.9	2.1 ± 0.5	2.7 ± 0.5	<3
3'/5' *trp*	1.9 ± 0.6	0.7 ± 0.1	1.3 ± 0.3	<3
5' Signal value bioB	1,562 ± 43	1,585 ± 65	1573 ± 39	Increasing stepwise trend (bioB, bioC, bioD, and cre)
5' Signal value bioC	4,660 ± 125	4,554 ± 125	4,607 ± 88	
5' Signal value bioD	8,458 ± 229	8,232 ± 242	8,345 ± 165	
5' Signal value cre	25,205 ± 730	24,492 ± 662	24,848 ± 490	

^a^Values are means ± SEM

#### Percent Present Call

The number of probe sets called "Present" relative to the total number of probe sets on the array is defined as a percentage present (%P), which is affected by cell/tissue type, RNA quality, environmental or biological stimuli, and probe array type. The percent present call should be >35% [31] and typically range between 35% and 65%. In our experiment from 42 chips, the mean %P was 65% with a range of 54.9–70.5% (Table 2). These findings indicate that the overall quality of RNA for the 42 arrays was high and reproducible.

#### Poly-A Controls: lys, phe, dap

The quality of the labeling process for the entire target can be monitored by Poly-A RNA. As described in the Affymetrix technical manual, "*Dap*, *lys*, *phe*, and *trp* are *B. subtilis* genes that have been modified by the addition of poly-A tails, and then cloned into pBluescript vectors, which contain T3 promoter sequences. Amplifying these poly-A controls with T3 RNA polymerase will yield sense RNAs, which can be spiked into a complex RNA sample, carried through the sample preparation process, and evaluated like internal control genes" [30]. The *dap*, *lys*, *phe* spikes should also have ratios <3. Spikes >3 indicate that there was likely a processing or reagent issue that effected the IVT reaction, whereas spikes <3 indicate successful processing. In our experiments, all of the 3'/5' ratios for the *lys*, *phe*, and *dap* were <3 (Table 2), which suggests that the sample processing was of high quality.

### 4.6 Hybridization Controls: bioB, bioC, bioD, and cre

As described in the Affymetrix technical manual, "BioB, bioC and bioD are genes in the *E. coli* biotin synthesis pathway. Cre represents the recombinase gene" [30]. These are pre-labeled spikes and can be used as an indicator of successful hybridization, washing, and staining. Anticipated results should demonstrate an increasing signal trend in the following order: bioB < bioC < bioD < cre. In our experiments, the increased trend of bioB, bioC, bioD, and cre in their signal values was observed (Table 2), which suggest that the hybridization, washing, and staining were successful and the efficiency of sample hybridization reached expectation.

## 5 Conclusions

The intent of this manuscript was to improve upon the original Histogene^®^ protocol for LCM by shortening incubation times and eliminating steps in the procedure. In addition, the high adipose content present in normal mammary epithelium presents a unique challenge in obtaining frozen sections suitable for LCM. Attention was focused on the preparation of material as well as timing at each stage and did not assume the proficiency level of the reader as is the case with many publications in the literature. One particular paper by Upson et al. [22] stands out and appears to be more focused on the comparison of amplification protocols and lacks detail in regards to preparation and timing of both tissue and LCM. In fact, no mention is made in "Materials and Methods" of any time-dependent issues related to RNA integrity, thus giving the reader a false sense of security regarding preparation and collection. MG and MA were rapidly excised and immediately snap-frozen in liquid nitrogen. MG was frozen as a whole mount preparation in order to maintain anatomical orientation and facilitate cryosectioning. Best results were obtained when cryosections were prepared in <20 min. Cryosections, 7 μm and 10 μm, respectively, for MA and MG, were processed without staining and epithelial and stromal cells were easily visualized on the LCM instrument via contrast adjustment of the microscopic image. Elimination of the staining step and subsequent water rinse in addition to decreased times for xylene clearing and air drying provided more time for LCM while maintaining adequate dehydration necessary for LCM and preserving RNA integrity. The use of PrepStrips™ to flatten prior tissue prior to LCM was helpful for both MA and MG sections. The digital tablet greatly reduced the time necessary for marking up AOIs for laser capture. Adhesive notes applied to the cap after LCM greatly aided in the removal of unwanted debris and did not appear to affect RNA integrity. Four LCM caps were required for MA (mean cell count per cap = 6,202.2 ± 223.2) and eight caps for MG sections (mean cell count per cap = 994.2 ± 42.5) to provide sufficient amounts of RNA for downstream microarray analysis (Table 1). The highest quality RNA was obtained when the entire LCM process was ≤30 min, i.e., from the point the slide was removed from dry ice and immersed in 75% EtOH until captured cells were placed in extraction buffer. The evaluation of quality control for all of the 42 gene chips indicated that the experimental procedures described in the current study provided high-quality RNA that can be used for gene array to generate high-quality data of gene expression. This optimized protocol will provide researchers facing similar challenges in the field of breast cancer research with a detailed and simplified approach to LCM that is reproducible and will yield high-quality RNA for downstream applications including microarray analysis.

## Abbreviations

AOI: Area of interest; DOA: Days of age; LCM: Laser capture microdissection; LN: Lymph node; MA: Mammary adenocarcinoma; MG: Mammary gland; NF: Nuclease free; OCT: Optimal cutting temperature; RIN: RNA integrity number; ROI: Region of interest; WB: Wash buffer
